# Core Histones Are Constituents of the Perinuclear Theca of Murid Spermatozoa: An Assessment of Their Synthesis and Assembly during Spermiogenesis and Function after Gametic Fusion

**DOI:** 10.3390/ijms22158119

**Published:** 2021-07-29

**Authors:** Lauren E. Hamilton, Morgan Lion, Luis Aguila, João Suzuki, Genevieve Acteau, Nicole Protopapas, Wei Xu, Peter Sutovsky, Mark Baker, Richard Oko

**Affiliations:** 1 Department of Biomedical and Molecular Sciences, Queen’s University, Botterell Hall, 18 Stuart Street, Kingston, ON K7L 3N6, Canada; hamiltonla@missouri.edu (L.E.H.); 12mrl3@queensu.ca (M.L.); gen.act2401@gmail.com (G.A.); 16nmp2@queensu.ca (N.P.); wx@queensu.ca (W.X.); 2 Division of Animal Science, College of Agriculture, Food and Natural Resources, University of Missouri, Columbia, MO 65211, USA; sutovskyp@missouri.edu; 3 Center for Research in Reproduction and Fertility, Department of Veterinary Sciences, Université de Montreal, St. Hyacinthe, QC J2S 2M2, Canada; luis.aguila.paredes@gmail.com (L.A.); jsuzukijr@yahoo.com (J.S.); 4 Department of Obstetrics, Gynecology and Women’s Health, School of Medicine, University of Missouri, Columbia, MO 65211, USA; 5 School of Environmental and Life Science, University of Newcastle, Callaghan, NSW 2308, Australia; Mark.Baker@newcastle.edu.au

**Keywords:** histones, gamete biology, spermiogenesis, microtubular manchette, spermatozoa, perinuclear theca, postacrosomal sheath, perforatorium, fertilization, ICSI, mass spectrometry

## Abstract

The perinuclear theca (PT) of the eutherian sperm head is a cytoskeletal-like structure that houses proteins involved in important cellular processes during spermiogenesis and fertilization. Building upon our novel discovery of non-nuclear histones in the bovine PT, we sought to investigate whether this PT localization was a conserved feature of eutherian sperm. Employing cell fractionation, immunodetection, mass spectrometry, qPCR, and intracytoplasmic sperm injections (ICSI), we examined the localization, developmental origin, and functional potential of histones from the murid PT. Immunodetection localized histones to the post-acrosomal sheath (PAS) and the perforatorium (PERF) of the PT but showed an absence in the sperm nucleus. MS/MS analysis of selectively extracted PT histones indicated that predominately core histones (i.e., H3, H3.3, H2B, H2A, H2AX, and H4) populate the murid PT. These core histones appear to be *de novo*-synthesized in round spermatids and assembled via the manchette during spermatid elongation. Mouse ICSI results suggest that early embryonic development is delayed in the absence of PT-derived core histones. Here, we provide evidence that core histones are *de novo*-synthesized prior to PT assembly and deposited in PT sub-compartments for subsequent involvement in chromatin remodeling of the male pronucleus post-fertilization.

## 1. Introduction

**Figure 1:**
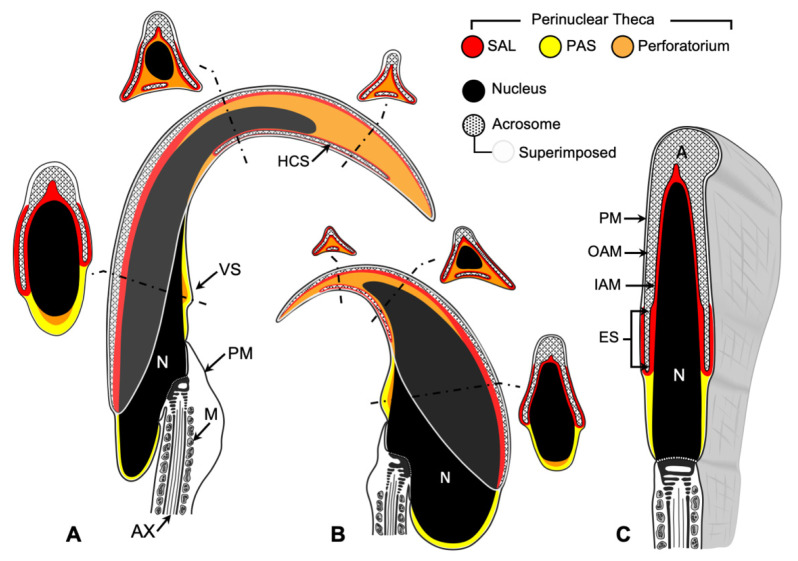
Diagrammatic representations of mid-sagittal sections through falciform (**A**) rat and (**B**) mouse sperm heads and spatulate (**C**) bull sperm heads. Regardless of morphological variations, the eutherian sperm head is comprised of three major cellular compartments: the nucleus (N; black), the acrosome (A; crosshatched pattern), and the perinuclear theca (PT; red/yellow/orange). The nucleus occupies the majority of the specialized sperm head volume. The acrosome overlays the apical half of the nucleus and is bound by the inner and outer acrosomal membranes (IAM and OAM, respectively). In the mid-sagittal sections of the murid sperm (**A**,**B**), the area that the acrosome covers is superimposed over each section for reference. The PT is formed by several continuous cytosolic compartments, which together encapsulate the entire condensed nucleus, except where the tail implants. The PT of spatulate sperm (**C**) is subdivided into the sub-acrosomal layer (SAL; red) and the post-acrosomal sheath (PAS; yellow). The equatorial segment (ES) is the thin caudal portion of the acrosome, covered on its outer surface by an extension of the SAL, the outer periacrosomal layer (OPL). In addition to SAL, OPL, and PAS regions, the PT of falciform sperm contains the perforatorium (orange), which extends apically from the nucleus to form the characteristic hook shape. The sperm tail is partially shown extending from the caudal nucleus. The core structure, the axoneme (AX), is surrounded by outer dense fibers, which in turn are surrounded by helically arranged mitochondria (M) in the midpiece of the tail. The plasmalemma (PM) encases the sperm head and tail. Labels: HCS, displaced head cap segment: VS, ventral spur. Adapted from Protopapas et al. [15], Oko and Maravei [16], and Lalli and Clermont [17].

There are three main compartments within the sperm head: the nucleus, the acrosome, and the perinuclear theca (PT) (Figure 1). The acrosome is a secretory vesicle that encapsulates the anterior region of the head and contains proteins required for oocyte penetration. Underneath the acrosome and surrounding the nucleus is the PT, a condensed cytosolic-protein-rich layer [1]. Based on compositional and functional considerations, the PT can be divided into two compartments: the subacrosomal layer (SAL), which houses proteins involved in the assembly and attachment of the acrosome to the spermatid nucleus during spermiogenesis [1,2,3,4,5,6], and the post-acrosomal sheath (PAS), which houses proteins implicated in oocyte activation and zygote development during and after fertilization [1,7,8,9,10,11,12,13,14]. In murids, the PT has an additional third compartment termed the perforatorium [15] (Figure 1). The perforatorium is a hook-shaped extension of the PT unique to falciform spermatozoa that encircles and extends beyond the apical nucleus. It is compositionally distinct from the SAL but has developmental, compositional, and functional similarities with the PAS [15].

The fraction of histones retained in the mature eutherian spermatozoon nucleus is species-dependent. The amount of histones found in the spermatid nucleus to date is also highly dependent on isolation and extraction techniques; however, in contrast to the differences in histone retention, there is minimal variability in the final stages of DNA packaging and compaction in eutherian species. Research to date has established a general model accounting for changes in nucleoprotein content during spermatogenesis, which in murids entails the sequential replacement of somatic-type histones by testis-specific variants, prior to the haploid phase, followed by smaller basic proteins commensurate with the increasingly compacted spermatid nucleus. It has been established in murids that prior to and during meiosis, somatic histones H1, H2B, H2A, and H3 are largely replaced by testis-specific histones [18]. More recently it has been shown that the gene encoding testis-specific histone variant H3t, which has a human counterpart, is essential for entry into spermatogenesis; its loss of function leads to azoospermia and infertility [19]. A testis-specific counterpart to core histone H4 has yet to be discovered with any certainty. Regardless, the core histones that reside in the spermatid nucleus post-meiotically are replaced by transition proteins in condensing spermatid nuclei [20,21,22], which are in turn replaced by protamines in late spermatids [23,24]. The consensus reached in most eutherian mammals is that the majority of basic proteins residing within the condensed mature sperm nucleus are protamines; however, a small amount of histones are retained in the mature sperm nucleus and have been shown to be resistant to ionic detergents used to isolate sperm nuclei [25,26,27,28] and extract perinuclear histones [29].

We have previously shown unprecedented non-nuclear localization of core histones in the PAS of bovine spermatozoa [29]. These histones were extractable from whole bull sperm or their isolated heads with ionic detergents or high-salt solutions; therefore, our first objective was to establish that this distinct core histone localization was not a unique feature of bovine spermatozoa, but rather a common feature of eutherian sperm. We chose the murid model to explore this possibility for several reasons. First, it has been the most commonly used model in studying the changes in nucleoprotein content during spermatogenesis. Secondly, velocity sedimentation techniques developed in murids to separate individual populations of seminiferous epithelial cells allowed us to address whether a new wave of histone synthesis occurs during spermiogenesis that provides core histones for PT assembly. Based on their localization and assembly during spermiogenesis [30], we hypothesized that the PT histones are synthesized *de novo* rather than recycled from the haploid nucleus after histone-replacement in condensing or elongating spermatids. This hypothesis is supported by the observation that hyperacetylation of histones immediately precedes and overlaps the process of nuclear histone replacement by transition proteins, and these acetylated histones are no longer observed in condensed spermatids, even after blocking deacetylase activity [31]. Thirdly, mouse intracytoplasmic sperm injection (ICSI) is an ideal technique to assess the consequences of inhibiting or depleting PT proteins on early zygotic development [13] because only the sperm nucleus and PT are required to achieve fertilization in the mouse via ICSI [8]; thus, we first explored the possibility of selectively extracting histones from the PT of the murid sperm head without disrupting oocyte activation. Once we established that this was possible, we assessed the consequences of PT histone depletion on early embryonic development in ICSI-fertilized mouse oocytes.

## 2. Results

### 2.1. Core Histones Are Constituents of the PAS and Perforatorium of the Rat PT

**Figure 2:**
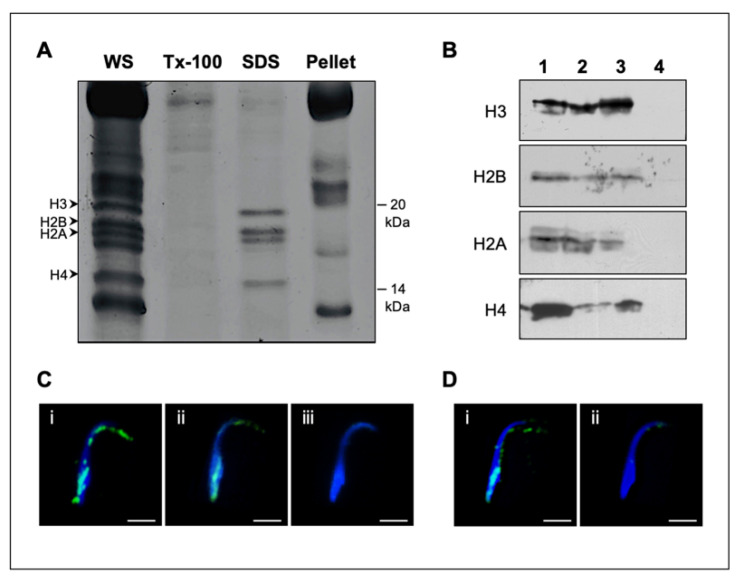
Histone extractability and localization in rat spermatozoa: (**A**) Coomassie gel profile of the four histones present within whole sperm (WS), their resistance to extractability with 2% Triton-100 (Tx-100), their extractability with 2% SDS (SDS), and the remaining unextracted bands within the pellet (Pellet); (**B**) the immunoreactivity of affinity purified histone antibodies against H3, H2B, H2A, and H4 with calf thymus histones (lane 1), rat whole sperm (lane 2), 2% SDS extract of rat whole sperm (lane 3), and the sperm pellet after SDS extraction (lane 4); (**C**) spermatozoa probed with an anti-pan histone antibody that reacts to all core histones (i), an H3 affinity-purified histone (ii), and Rabbit IgG control (iii), all merged with DAPI nuclear labeling; (**D**) TX-100 (i) and SDS (ii)-extracted spermatozoa probed with anti-H3 antibody and merged with DAPI labeling. White bar = 5 μm.

The presence of all four core histones within the perinuclear theca of the sperm head was first discovered in bull spermatozoa [29]. To explore whether they are present in rat spermatozoa, protein profiles of non-treated, 2% Triton-X-100 (TX-100)-extracted, and 2% SDS (SDS)-extracted rat whole sperm (WS) were run using 18% SDS-PAGE gel electrophoresis under reducing conditions and stained with Coomassie Brilliant Blue 250 (Figure 2A). Four bands in the range of 14–20 kDa were identified and determined to be resistant to extraction with non-ionic detergent (TX-100) but fully extractable with ionic detergent (SDS), suggesting that they were ionically bound. Verification of their identity as histones was accomplished by probing with affinity-purified antibodies specific for each of the core histones on Western blots containing purified calf thymus core histones (Figure 2B, lane 1), whole rat spermatozoa (Figure 2B, lane 2), SDS extract of whole rat sperm (Figure 2B, lane 3), and the SDS-extracted sperm pellet (Figure 2B, lane 4). Importantly, each of the four immuno-reactive calf thymus histones corresponded to only one of the four immunolabeled whole sperm or SDS-extracted bands (Figure 2B, lanes 2, 3), and no immunoreactivity was evident in the extracted sperm pellet (Figure 2B, lane 4). This indicated that the four bands represent the four core histones.

**Figure 3:**
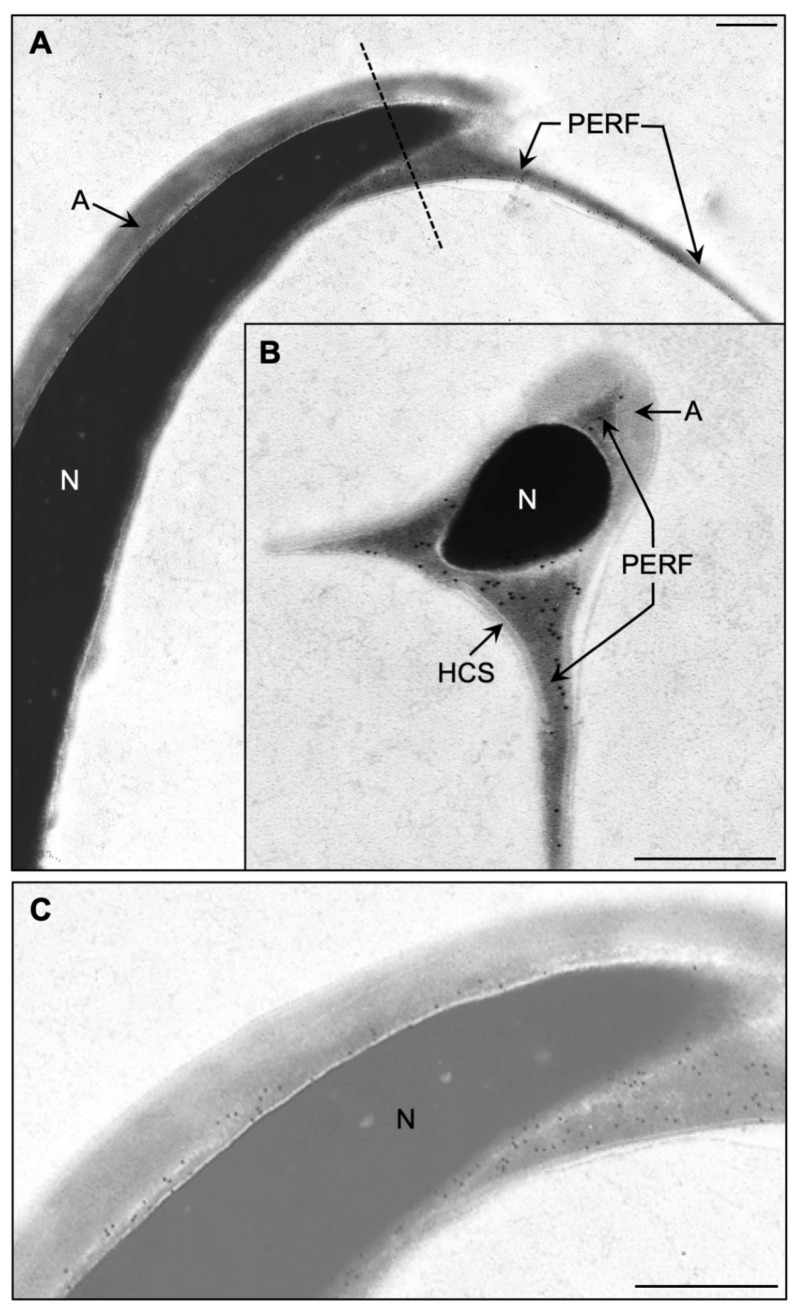
Transmission electron microscopy showing the presence of histones in the PT of cauda epididymal rat sperm: (**A**) A sagittal section through the sperm head labeled with an affinity purified anti-H3 antibody; (**B**) a coronal or cross section through the apical perforatorial region (in the area shown by the dashed line in (A) labeled with anti-H3 antibody (note that all three prongs of the perforatorium are immunogold-labeled); (**C**) reduced exposure of negative to show that the nucleus in contrast to the PT is not immunoreactive to the anti-histone antibody. Labels: A, acrosome; N, nucleus; PERF, perforatorium; HCS, displaced head cap segment. Bars = 1 μm.

To determine the localization of these histones, rat spermatozoa were probed with an anti-pan core histone immune serum. Fluorescent immunoreactivity was detected in the PAS of the sperm head, as previously shown in the bull, and additionally in the perforatorium (Figure 2C, i), a structure that is not present in bull sperm. The specificity of this localization was tested by utilizing an affinity-purified anti-H3 antibody, which abeled identically to the anti-pan histone antibody (Figure 2C, ii). Immunofluorescence, using the pan-histone antibody, also verified that SDS extraction (Figure 2D, ii), in contrast to TX-100 extraction (Figure 2D, i), removed most of the core histones from the PT (Figure 2C, i,ii). Immunogold labeling with the affinity-purified anti-H3 antibody at the electron microscope (EM) level confirmed the perforatorial immunoreactivity (Figure 3A), displaying reactivity within all three prongs of the perforatorial region of the PT (Figure 3B). Decreased exposure of the electron micrograph negatives showed negligible nuclear immunogold labeling when compared to labeling of the PT (Figure 3C), suggesting that most of the core histones in spermatozoa reside in the PT. 

### 2.2. KCl Extraction Selectively Depletes the Murid PT of the Core Histone Content

**Figure 4:**
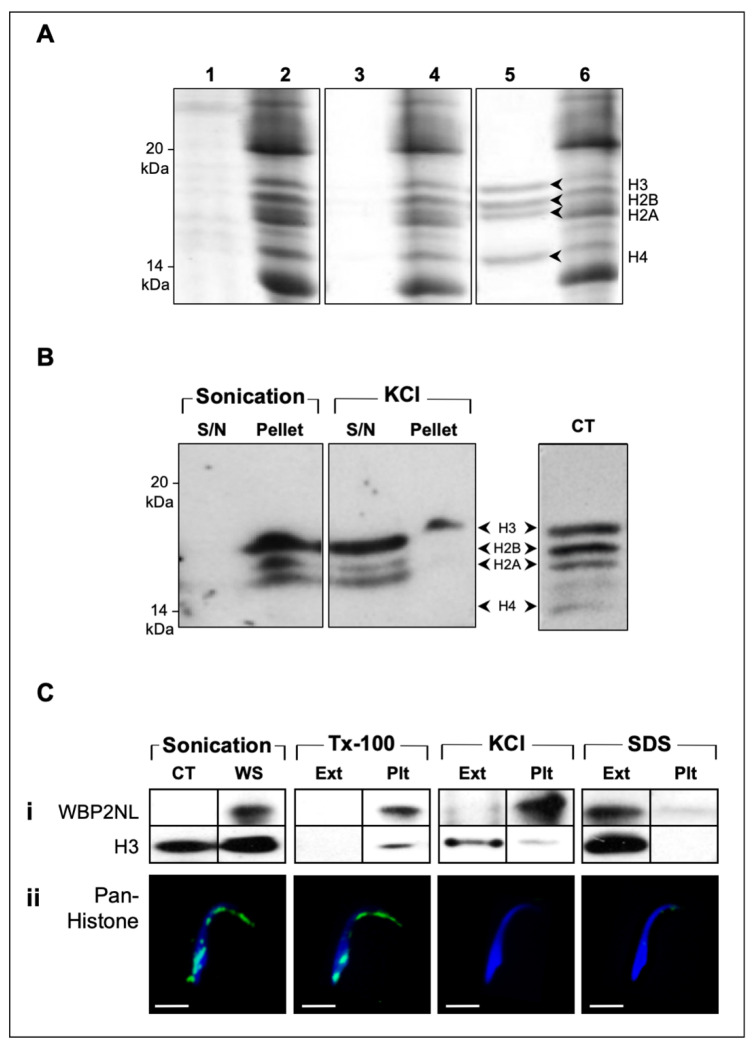
SDS-PAGE, immunoblot and immunofluorescence analyses of protein extracts from whole rat spermatozoa: (**A**) Coomassie-stained, 16.5% gel displaying four prominent protein bands extracted from rat whole sperm by mild sonication in 1 M KCl (lane 5), with corresponding profiles of solubilized sperm pellet before and after high-salt extraction (lane 6); conversely, these bands were not visible in Triton X-100 (lane 1) or sonicated (lane 3) extracts of rat whole sperm relative to corresponding pellets (lanes 2 and 4, respectively); (**B**) immunoblot probed with anti-pan histone antibody revealed consistent labeling patterns between calf thymus-derived histones (CT) and 1 M KCl extract of whole rat sperm; minimal labeling was detected in the KCl pellet following high-salt extraction, only a remnant of H3 band was discernable (s/n, supernatant); (**C**) (i) immunoblots of sperm extractions in 2% Triton-X100, 1 M KCl, and 2% SDS probed with an affinity-purified H3 antibody to confirm histone extractability and an anti-WBP2NL/PAWP antibody to assess perinuclear theca extraction selectivity; (ii) sperm extracts from each extraction pellet were also analyzed by indirect immunofluorescence using an anti-pan histone antibody to examine the efficiency of each extraction and the integrity of the head shape after each treatment. CT, calf thymus-derived histones; WS, whole sperm; Ext, extract; Plt, pellet. White bar = 5 μm.

All four core histones within the bull sperm head were both SDS- and KCl-extractable [29]; therefore, we tested their extractability in rat spermatozoa (Figure 4) under the latter condition. Selective extraction of the PT histones was critical in our analysis of their sequence identity by mass spectrometry (MS) and in determining the consequences of PT histone depletion on early embryonic development. In order to make the 1 M KCl extraction more time-efficient and less prone to protease activity, sperm were sonicated in 1 M KCl on ice for 5 s at 0.1 A, then centrifuged to separate the supernatant (Figure 4A, lane 5) and pellet (Figure 4A, lane 6). In contrast to the four prominent bands present in the KCl extract (Figure 4A, lane 5), sonication alone (Figure 4A, lane 3) or Triton X-100 incubation (Figure 4A, lane 1) were shown to be ineffective in obtaining this histone banding pattern (Figure 4A).

Immunoblots probed with an anti-pan histone antibody confirmed the identity of the four prominent KCl-extracted bands as core histones (Figure 4B). In agreement with SDS-PAGE results, KCl supernatant (s/n) extracts displayed considerable immunoreactivity, especially in protein bands corresponding to H3, H2B, and H2A. Differences in antibody labeling intensity across the four polypeptide bands were attributable to differences in the rates at which individual histones transferred to Western blots, as well as to their individualistic antigenic responses. Noticeably, H3 readily transferred and elicited a high degree of antigenicity to the pan-histone antibody, whereas H4 was far more resistant to transfer and elicited a weaker signal. Nonetheless, moderate immunolabeling of the latter band was achieved with prolonged film exposure. In contrast, extracts from treatment with sonication only resulted in no detectable antigenicity, with all immunoreactivity being observed in the pellet (Figure 4B).

As expected, high-salt (1 M KCl) treatment was the most histone selective of the four PT extractability treatments (sonication, 2% Tx-100, 1 M KCl, and 2% SDS), as demonstrated by its inability to extract its PT neighbors and candidate oocyte-activating factor, WBP2NL/PAWP (Figure 4C, i). The KCl extractability of the non-nuclear histones confirms that they are anchored to the PT by ionic bonds, while most PT proteins are thought to be covalently attached and require harsh solubilizing conditions to detach them from this structure. Furthermore, since salts are impermeable to membranes, the vast majority of KCl-extractable histones likely arise from the PT rather than the sperm nucleus, whose nuclear envelope remains intact after this treatment [29]. To confirm that the biochemical extractions of the histones matched their absence in the PT of the sperm head, immunofluorescent localization was performed before and after extractions on rat spermatozoa using the anti-pan histone antibody (Figure 4C, ii). With the exception of Tx-100 treatment, all extraction procedures eliminated the majority of the histone immunoreactivity from the sperm head, barring a vestige of reactivity in the perforatorium. Furthermore, based on the DAPI nuclear staining, no extraction protocols appeared to impact the shape or density of the sperm nuclei.

### 2.3. KCl Extraction of Murid and Bovine PT Histones for Comparative MS Sequence Analysis

**Figure 5:**
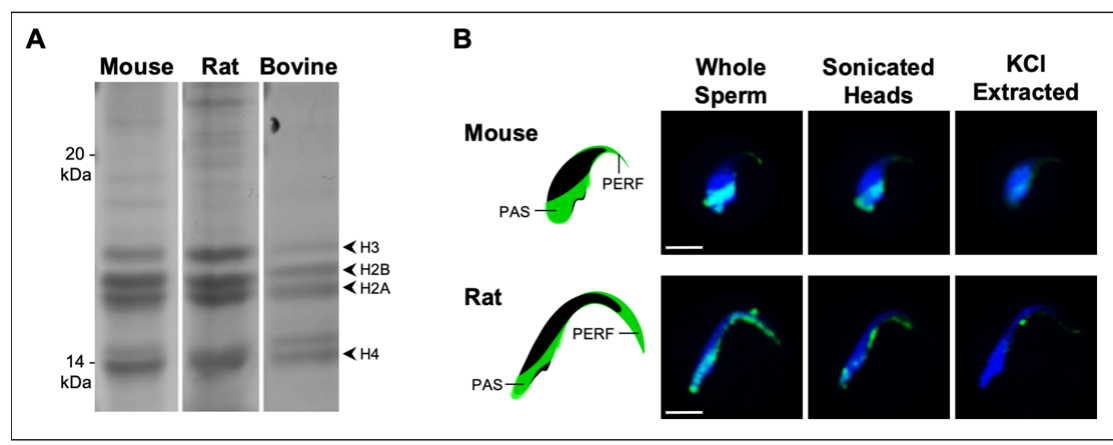
Comparison of KCl extracts between murid and bovine sperm and localization of core histones in murid PT before and after KCl treatment: (**A**) Coomassie-stained PAGE gel displaying KCl protein extracts from mouse, rat, and bovine whole sperm. Sperm were sonicated and washed before KCl extraction to remove acrosomal contaminants. The banding patterns of the four core histone sub-units were consistent across all three species, indicating that the KCl extraction method is effective in various sperm head morphologies. (**B**) Immunofluorescent labeling of mouse and rat sperm (Whole Sperm), with an anti-pan histone antibody localizing core histones (green) to the PAS and PERF regions of the PT. The diagrammatic mouse and rat sperm heads demonstrate the labeling of the anti-pan histone antibody in the PAS and PERF regions (green). Labeling was drastically decreased in sperm from both murid species following mild sonication in 1 M KCl (KCl extracted), with a vestige of core histone content persisting in the PERF region. Sonication (sonicated heads) did not appear to significantly reduce labeling of PT core histones relative to untreated whole sperm. DAPI labeled the nucleus (blue). Refer to Figure S1 for more detail regarding immunolocalization of histones in mouse sperm. Bar = 5 μM.

**Figure 6:**
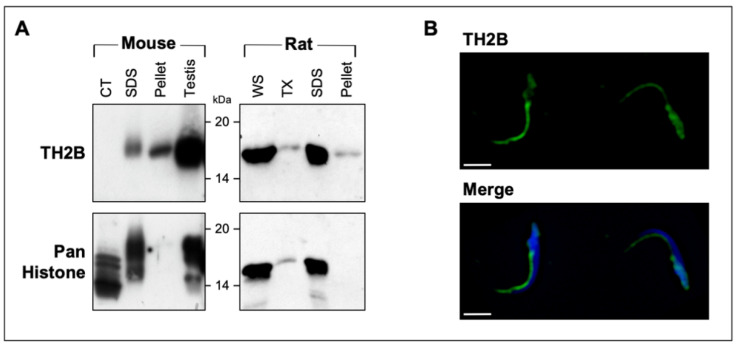
TH2B extractability and localization in murid spermatozoa: (**A**) Comparison of immunoreactivity between anti-TH2B and anti-pan histone antibodies on immunoblots of calf thymus histones (CT), 2% SDS extraction of spermatozoa (SDS), the sperm pellet after extraction (pellet), whole sperm (WS), 2% TX-100 (TX) extraction of spermatozoa, and testis homogenates (testis). According to the immunolabeling, anti-TH2B does not recognize somatic histones and is found in both SDS extracts and the resulting sperm pellet, indicating that it resides in the perinuclear theca and sperm nucleus, respectively. KCl extraction (not shown) showed similar results to SDS extraction. (**B**) Immunofluorescence confirmed TH2B’s presence in both the PAS and perforatorial regions of the PT. Bar = 5 μM.

To ensure that the KCl-extracted histones were derived from the PT and to lower the risk of contamination from sperm proteins residing in other sperm compartments, sperm were sonicated, centrifuged, and washed before KCl extraction. Sonication prior to KCl extraction removes the plasmalemma and the acrosome but keeps the PT and the nuclear membrane intact. Preservation of the nuclear membrane prevents salt from penetrating into the nucleus and inhibits the leaching of any residual nuclear histones during the extraction. These KCl extracts show almost identical profiles of PT histones in murid (mouse, rat) and bovine spermatozoa (Figure 5A). Correspondingly, the KCl-extracted sperm show marked decreases in histone reactivity in the PAS and PERF regions of the PT when viewed by immunofluorescence (Figure 5B). Table 1 compares the LC-MS/MS sequence analyses of the PT histones in mouse, rat, and bull models. A general conclusion from these analyses is that predominately core histones (i.e., H3, H3.3, H2B, H2A, H2AX, and H4) populate the murid PT along with variants TH2B and H2AV and canonical H1 linker–histone subtypes. Utilizing a specific anti-TH2B antibody, we were able to confirm that TH2B is also a resident of the PAS and PERF regions (Figure 6).

MS/MS of KCl-extracted PT histones showed post-translational modifications (PTMs) on H1.1, H2B type 2-B, H3.1, H3.3, and H4 in mice and H3.1 and H3.3 in rats (Table 2). No PTMs were associated with H2A in mice or rats, in agreement with previous reports finding low levels of H2A modification sites in somatic cells [32].

In mice, two acetylation modifications (H3K18ac, H3K23ac) were found in both H3.1 and H3.3, with two additional sites of acetylation only identified in H3.3 (H3K27ac, H3K79ac). Methylation modifications on H3 were also uncovered on known sites of gene regulation such as K27, a site with high rates of trimethylation that has been shown to mark developmental regulators, affect sperm function, and influence embryo development in various species [33,34,35]. Interestingly, while H3K27me3 was found in both H3.1 and H3.3 in rats, only dimethylation of residue K27 was found in mice. Moreover, solely mono- and dimethylations were found in the KCl extracted PT histones in mice and predominated the methylation modifications in rats. This preference for mono- and dimethylation is thought to be a signature in sperm histones but its significance is unknown [36]. Furthermore, we also discovered that H3K36me2 was only found on peptides that also had H3K27me2 modifications in mouse H3.3, a phenomenon that was previously reported in mouse spermatozoa and suggestive of H3 crosstalk between the two residues [36].

Two PTMs (H4K20me, H4R23me2) associated with PT H4 in mice have been previously reported in mature mouse spermatozoa. H4K20me has been implicated in chromatin compaction and transcriptional repression, with H4R23me proposed to act as a possible regulator of H4K20me interactions with its potential binding partners [37]. These epigenetic markers were not seen in rats. Importantly, the H4K16ac modification, a critical signal and target for degradation through a polyubiquitination-independent pathway, which was added in the nucleus during the histone–protamine transition, was absent in both our mouse and rat PT histones.

### 2.4. The Developmental Origin of the Non-Nuclear Histones during Spermiogenesis

**Figure 7:**
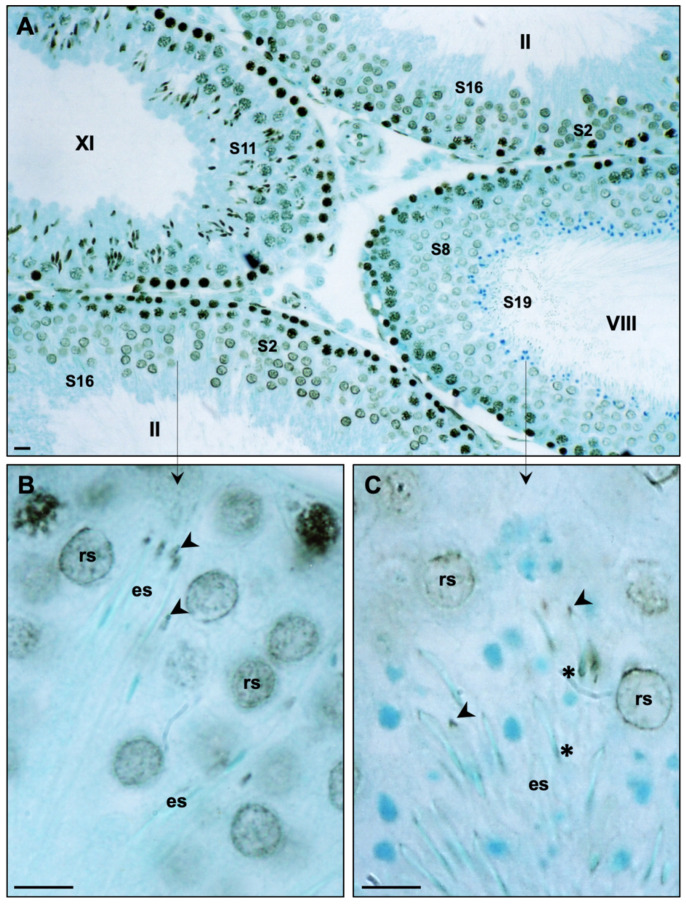
Light micrographs of immunoperoxidase-labeled sections, counterstained with methylene blue, through rat seminiferous tubules of stages II, VIII, and XI probed with an affinity-purified anti-H3 antibody: (**A**) Immunoreactivity of varying intensity is found in the nuclei of most germ cells, except for elongated spermatids (es) found in stages II and VIII. Bar = 20 μm (**B**,**C**). Arrows from stages II and VIII in (**A**) point to a similar region (not identical) of the seminiferous epithelium, magnified several-fold, where elongated spermatids reside. Note that the tips or perforatoria of spermatid heads are immunoreactive (arrowheads). (**C**) The triangular rod-like structure of the perforatorium (arrowheads) is made evident by the immunoperoxidase labeling. In some planes of the section, immunoreactivity in the caudal part of the elongated head or post-acrosomal sheath region is evident (asterisk). Bar = 5 μm; rs, round spermatid; S2–S19, spermatid steps 2 to 19 of spermiogenesis. Anti-histone labeling of the post-acrosomal sheath can be seen more prominently in Figure S3C.

Testicular sections probed with anti-pan histone and anti-H3 affinity-purified antibodies yielded identical patterns of immunoperoxidase localization; these patterns of immunoperoxidase reactivity will be collectively reported as anti-histone labeling. As expected, anti-histone labeling in rat testicular sections was observed in the nuclei of all somatic and most germ cells of the seminiferous epithelium, with the exception of elongating spermatids that had undergone nuclear protein transitions from histones to transition proteins (TP1 and TP2) to protamines (Figure 7). Spermatid nuclear condensation and elongation was associated until step 11, with an increase in the intensity of histone immunoreactivity within the nucleus. This was followed by a steady decline, so that by steps 13 and 14 little histone labeling was seen in the spermatid nucleus (Figure 7 and Figure S2). In steps 15 and 16, well after the bulk of nuclear histones were replaced by basic proteins, anti-histone reactivity was found associated with the apical portion of the developing sperm head (Figure 7B), which by step 19 was clearly associated with the triangular pronged structure of the PERF region (Figure 7C and Figure S3A,B). In early step 16, anti-histone immunoreactivity was first seen associated with the caudal part of the spermatid head in the region of the PAS (Figure S3C). Anti-histone reactivity in both the PERF and PAS regions of the PT was evident in step 19 in spermatids being released from the seminiferous epithelium into the tubular lumen (Figure 7C and Figure S3A–C). The deposition of histones into the developing mouse PT (Figure S4) followed a similar pattern as described above in the rat.

**Figure 8:**
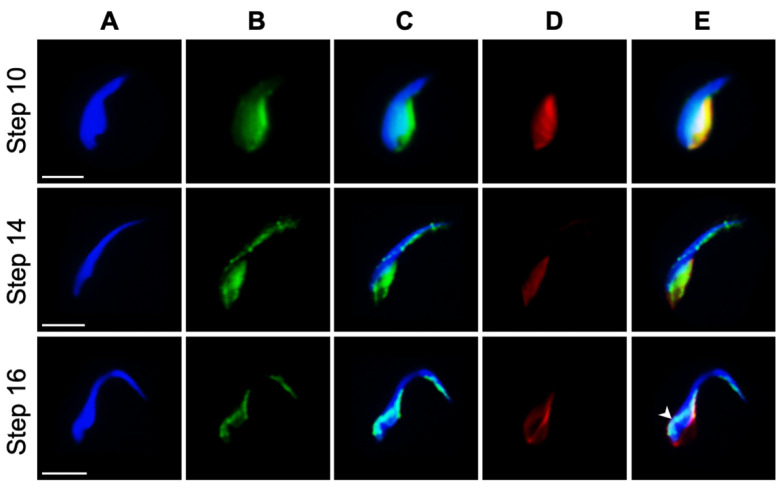
Immunofluorescent micrographs of the co-localization of the pan-α-histone and α-tubulin antibody labeling on the caudal manchette of rat spermatids. Rat spermatids from testicular extracts were fixed in 2% formaldehyde and permeabilized after fixation with Triton-X-100: (**A**) DAPI alone; (**B**) anti-histone antibody alone; (**C**) panels A and B merged; (**D**) anti-tubulin antibody alone; (**E**) panels A, B, and D merged. Note in step 14 the histones are already deposited apically into the perforatorial region of the spermatid head, while in step 16 the histones assemble as part of the PAS (arrowhead) in the wake of the descending manchette. Blue = DAPI, green = anti-pan histone, red = anti-tubulin, yellow = co-localization of the histones and tubulin. White bar = 5 μm. Also see Figure S5.

Past investigations into the mode of PAS protein deposition shows that these proteins ascend from the cytoplasmic lobe of the spermatid into this PT sub-region via the microtubular manchette [12,30,38]. Therefore, we investigated the relationships between the microtubular manchette and the non-nuclear histones through fluorescent co-localization in extracted spermatogenic cells of the rat testes (Figure 8). Non-nuclear histones are seen co-localized with the microtubules of the manchette in the elongation phase of spermiogenesis, even before the nuclear histones are expelled from the spermatid nucleus (Figure 8, step 10). Moreover, after nuclear expulsion of the histones, the non-nuclear histones begin to be channeled into the perforatorial region of the spermatid head via the manchette (Figure 8, step 14). Later, from late step 15 to early step 16 of spermiogenesis, the manchette descends along the caudal half of the spermatid nucleus, signifying the end of the elongation phase. In the wake of the manchette’s descent, the histones that are transported along its microtubules appear to assemble as part of the PAS (Figure 8, step 16). By late in step 16, the PAS is fully formed and the manchette completely detaches from the sperm head and deteriorates in the cytoplasmic lobe of the spermatid. This close association of histones with the manchette was also evident during swine spermiogenesis (Figure S5).

### 2.5. The Origins of Core Histones Residing in the PT

To investigate whether histones destined for PT assembly could be *de novo*-synthesized during spermiogenesis, real time-PCR was performed on equal numbers of isolated haploid (predominately round spermatids) and tetraploid (predominately pachytene spermatocytes) cells using a pair of primers specific for core somatic histone H2B mRNA. Based on cell number, our preliminary findings showed that the H2B mRNA expression level or copy number was only 1.5 times higher in tetraploid cells when compared to haploid cells, even though the latter germ cells were on average 2.4 times larger than the former. Most importantly, however, when the mRNA copy number was calculated based on DNA quantity within each cell type, the haploid cell had 2.6 times more somatic H2B per DNA than the tetraploid cell (Figure S6). The greater expression of core somatic histone H2B mRNA during the round spermatid stage, coupled with the fact that testis-specific H2B (TH2B) is the predominant H2B-type histone in the nucleosome of spermatocytes and round spermatids [39], suggests that *de novo* production of histones occurred during the final opportunity for transcription prior to nuclear spermatid compaction.

### 2.6. PT Histone Deficiency in Mouse Sperm Delays Early Embryonic Development

Mouse zygotes were ICSI-generated using 3 different sperm treatment groups: untreated, motile whole sperm (ICSI CTRL); sperm that were sonicated in PBS to account for the isolated effect of sonication (SON CTRL); and our experimental PT histone depleted (HD) sperm, which were sonicated in high salt (1 M KCl).

**Figure 9:**
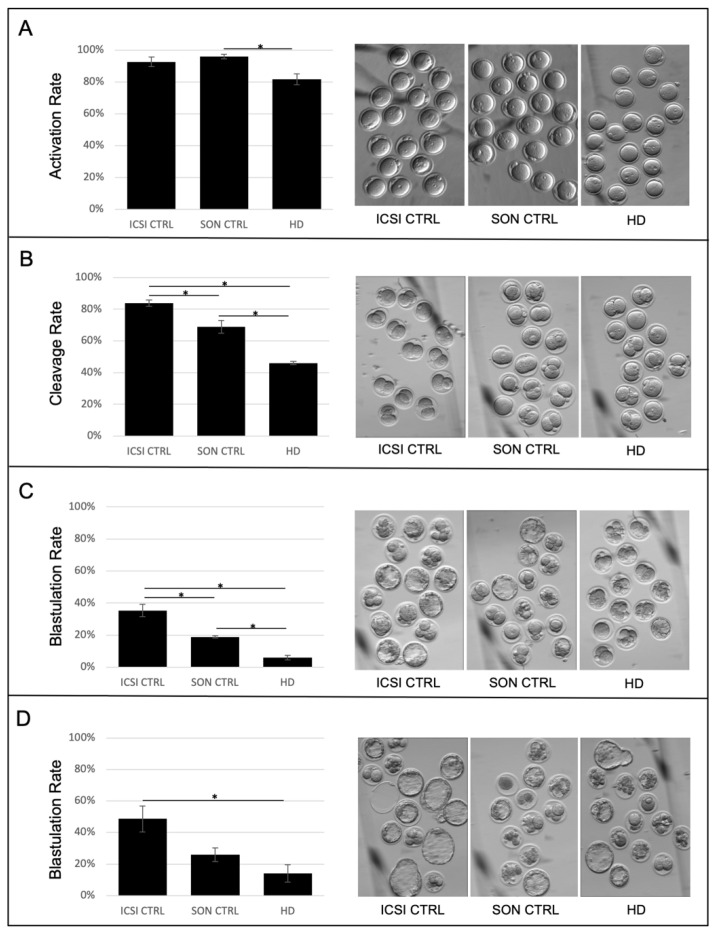
Mouse embryo development of ICSI-injected oocytes fertilized with PT-histone-deficient spermatozoa: (**A**) Oocytes injected with PT histone deficient (HD) spermatozoa activate at a rate comparable to controls. The number of activated oocytes in each experimental group was counted at 6 h post-ICSI and four replicates were performed per experimental group. The activation rate of HD oocytes (81.7%, *n* = 91) was significantly lower than that of SON CTRLs (96.0%, *n* = 95), but not significantly different from ICSI CTRLs (92.6%, *n* = 81). (**B**) HD zygotes cleave at a lower rate than controls. The number of cleaved embryos in each experimental group was counted at 22–24 h post-ICSI and four replicates were performed per experimental group. The cleavage rate of HD zygotes (46.0%, *n* = 91) was significantly lower than that of ICSI CTRL (83.8%, *n* = 81) and SON CTRL (68.9%, *n* = 95) groups. Control groups also displayed significantly different rates. (**C**) HD embryos blastulate at lower rate than controls on day 4. The number of blastocysts in each experimental group was counted at 4 days post-ICSI and three replicates were performed. The blastulation rate of HD zygotes (6.0%, *n* = 58) was significantly lower than that of ICSI CTRL (35.3%, *n* = 62) and SON CTRL (18.9%, *n* = 69) groups. (**D**) HD embryo blastulation rates improve as development progresses. The number of blastocysts in each experimental group was counted at 5 days post-ICSI and three replicates were performed. The blastulation rate of HD zygotes (14.1%, *n* = 58) was significantly lower than that of ICSI CTRL (48.7%, *n* = 62) but not statistically different than SON CTRL (25.9%, *n* = 69). For all panels *p* values ≤ 0.05 are denoted by a star (*), error bars represent SEM and representative images of each group are shown.

The activation rate of each group was investigated at 6 h post-injection, and embryonic development was further assessed at both the first cleavage (22–24 h post-injection) and blastocyst formation (96 h post-injection) stages. Whilst activations rates between the HD (82%) and SON CTRL (96%) groups were significantly different (Figure 9A), there were no significant differences between HD and ICSI CTRL(92%) (Figure 9A) or SON CTRL and ICSI CTRL groups (Figure 9A). The comparable activation rates between the HD and control groups suggests that the high-salt extraction methodology employed to selectively extract the PT-bound histones was selective enough to preserve other essential PT residents such as sperm-oocyte-activating factors (SOAF).

Zygotes were further assessed at first cleavage (22–24 h post-injection). At this timepoint, 46.0% of HD zygotes achieved the 2-cell stage; a significantly lower average cleavage rate than that observed in either control group (ICSI CTRL, 83.8% and SON CTRL, 68.9%) (Figure 9B). Blastocyst formation was also assessed 4 days post-injection and the rate of blastulation was determined as the percentage of the total number of oocytes injected that reached at least early blastocyst stage and contained a blastocoelic cavity by 96 h. By 4 days, 6% of HD zygotes had become blastocysts; a significantly lower rate of blastocyst formation than that observed in either the SON CTRL (19%) or the ICSI CTRL (35%) (Figure 9C); however, if permitted to continue developing until day 5, HD embryos showed the ability to recover to a blastocyst rate (14%) that was not significantly different from the sonicated control (SON CTRL)(26%), but was still significantly lower than the ICSI control (ICSI CTRL)(49%) (Figure 9D).

## 3. Discussion

We report unequivocal evidence for the non-nuclear localization of core histones and some of their variants in mature murid spermatozoa. As was found in bull spermatozoa, these non-nuclear histones assemble as part of the postacrosomal sheath (PAS) of the perinuclear theca (PT) in murids but additionally assemble as constituents of the perforatorium (PERF). As recently investigated, the perforatorium is an apical extension of the PT, found in falciform spermatozoa of murids, which shares developmental and compositional similarities with the PAS [15]. It should be emphasized that the histones assembled in the perinuclear theca make up the majority of the histone content in both the murid and bovine sperm heads to the extent that after high-salt extraction of the PT histones, little to no antigenic traces of histones can be found in the extracted sperm heads, even though their nuclei remain intact; therefore, investigators exploring solely nuclear histones in spermatozoa should take precaution to extract the PT histones, either with an ionic detergent or high salt buffer, from the sperm head before attempting compositional analysis and histone immunolabeling studies of the sperm nucleus. Our findings suggest that the non-nuclear histones found within the perinuclear theca are conserved constituents within eutherian sperm.

Our mass spectrometric (MS) analysis of PT-derived histones suggested that predominately core histones populate the PT of bovine and murid spermatozoa (see Table 1). This compositional analysis was supported by comparative 2D acid–urea–Triton-SDS-PAGE coupled immunoblotting analysis of bull PT and calf thymus histones performed previously [29]. It was almost certain from our MS analysis in both murid and bovine that PT constituents H4 and H3 are core histones that are not made up of testis-specific variants. This is not surprising as a testis-specific counterpart to core histone H4 has yet to be discovered [40,41] and murid testis-specific H3 (H3t), which has a human counterpart (TH3), is absent in mature mouse spermatozoa, while core H3s (H3.1–3), including somatic variant H3.3, are present [19]. This could suggest that nucleosomal H3t, which is essential for spermatogonial differentiation and entry into meiosis [19], is replaced by somatic H3 (H3.1-3) during the round haploid phase of spermiogenesis at the same time that H3 (H3.1-3) synthesis is directed for PT assembly for spermatid elongation. H3.3, which differs from H3.1 by 5 amino acids, can be incorporated into the nucleosome both coupled to and independent of replication and provides a mechanism for the immediate activation of gene transcription [42,43].

Analysis of the post-translational modifications (PTMs) on PT-derived histones from both mouse and rat spermatozoa did not show widespread acetylation, indicative of the hyperacetylated state of histones prior to nuclear expulsion, but rather higher rates of methylation (Table 2). Additionally, as previously stated the H4K16ac modification, a critical signal and target for degradation through a polyubiquitination-independent pathway added in the nucleus during the histone-protamine transition was absent in both our mouse and rat PT-derived histone populations. Most PTMs discovered in the PT-derived histones of both mice and rats had previously been reported in spermatozoa and were present on residues implicated in transcription regulation, such as K18, K23, and K27 of histone H3 (Table 2) [33,34,35]; however, one PTM of interest, H3R26me2, found in mice has been reported to be an asymmetrical epigenetic modification between maternal and paternal genomes within the early embryo [44] and has been speculated as a possible marker for pre-patterning in the pre-implantation embryo, as blastomeres with higher H3R26me2 levels show preferential sorting into the inner cell mass [45]. To date, the origin of the H3R26me2 modification in the paternal genome is unknown but its presence on PT-derived histones may add further support to our hypothesis that the PT histones are not recycled from the spermatid nucleus but rather destined for post-fertilization events.

The relatively high expression of core somatic H2B mRNA found in the isolated rat round spermatids as compared to spermatocytes suggests that its translational product may be destined for PT assembly during the elongation phase of spermiogenesis. This is due to the fact that there appears to be no need for somatic H2B in the maintenance of the spermatid nucleosome, since testis-specific H2B (TH2B) is the predominant H2B-type histone of the round spermatid murid nucleosome, playing a key role in the histone-to-protamine transition of the mouse male genome [39]; therefore, we hypothesize that the excess production of this histone is transported to and compartmentalized within the PT. This does not exclude TH2B from participating in PT assembly, since immunoblots of PT extracts of murid spermatozoa, utilizing a specific anti-TH2B antibody, as well as mass spectrometry of PT extracts, indicate that TH2B is a constituent of the PT in murids and that a portion of TH2B may even be retained by the nucleus. We are uncertain whether the PT portion of TH2B arises from *de novo* synthesis in haploid spermatids or from recycled histones during the nucleosomal–protamine transition; however, there are two lines of evidence that support the former hypothesis. First, it appears that most histones removed from the nuclei of condensing spermatids are discarded and degraded [31]. Secondly, by injecting, tracking, and comparing intra-testicular ^3^H-lysine, -arginine, and -thymidine over time in various isolated germ cell populations, Goldberg et al. [46] demonstrated that histones (H1, H3, H2B, H2A, and H4) are synthesized *de novo* in elongating spermatids, becoming an integral part of the spermatozoa. An inherent weakness in this investigation was that there was no accounting for a PT source of histones, as spermatid and spermatozoa “nuclei” were isolated for analysis only in the presence of non-ionic detergents.

Our MS analysis of PT extracts from bovine spermatozoa was unable to detect TH2B. The reason for this could be that as far as we are aware, testis-specific histones have not been found in bovine models. Nevertheless, it appears that somatic H2B and TH2B may be interchangeable in murids; gene ablation of *Th2b* in the mouse model was shown to have no phenotypic consequences on spermatogenesis or fertilization and somatic H2B levels were significantly increased in spermatogenic cells in the absence of TH2B, suggesting compensation [39]. In addition, there is some question as to whether TH2B should even be regarded as testis-specific, as it is also detected in metaphase II mouse oocytes, in paternal and maternal pronuclei, and in polar bodies in one-cell mouse embryos [39]. In oocytes, the source appears to be the maternal genome, as TH2B is absent in embryos resulting from wild-type males and TH2B-less females.

In the H2A mammalian family, 10 genes encode for conventional or core histone H2A1 types, several genes encode for core histone H2A2 types, and 6 genes encode for histone variants H2AX, H2AZ, macroH2A1, macroH2A2, H2A-Bbd, and H2AV [43,47,48] (UniProt Search, https://www.uniprot.org (accessed on 7 April 2021)). Although H2A core histone types H2A1 and H2A2 appeared to prevail in the murid and bovine sperm PT, variants H2AV showed up in the PT of both families, while variant H2AX was found in murid sperm alone (Table 1). H2AV may be essential for heterochromatin formation and repression of transcription [48], while H2AX, which is 95% similar to conventional H2A, may facilitate the repair of chromosomal double-strand breaks when phosphorylated [47,49].

It is doubtful that the energy spent to synthesize and assemble the core histones as part of the PT at the end of spermiogenesis would not lead to a functional purpose. The fact that soon after sperm–oocyte fusion the PAS region of the PT begins to rapidly solubilize in the oocyte cytoplasm and release proteins essential for oocyte activation [9] and decondensation of the sperm chromatin [13] suggests that the histones released from the same compartment would also contribute to the fertilization process. Given their inherent ability to bind and protect DNA and regulate its activities, we hypothesized that PT-associated histones, once released in the oocyte cytoplasm, stabilize decondensing sperm chromatin during paternal pronuclear formation in the developing zygote until oocyte or zygote produced histones are able to take over. If this were true, one would expect that zygotes fertilized with PT histone-deficient sperm would experience a delay in early developmental events as the paternal pronucleus attempts to recover from the effects of chromatin instability. In agreement with this idea, developmental delay was observed in zygotes fertilized with PT-histone-depleted spermatozoa as early as the two-cell stage. This delay continued to be prominent into blastocyst stage, suggesting that destabilization of the male chromatin has a prolonged effect on the developing embryo. This interpretation warrants precaution as the possibility exists that the KCl extraction could also remove yet unknown PT-protein entities, in addition to the histones, which could contribute to the embryonic delays observed; however, our lab has not been able to identify any other PT proteins that are KCl-extractable. Furthermore, as far as we are aware, besides WPB2NL/PAWP [7,10] and GSTO2 [13], which are not KCl-extractable, no other sperm PT proteins have yet been shown to be required for fertilization utilizing the ICSI process.

As the embryo transitions through the mitotic cell cycle, checkpoints ensure the completion of phase-specific tasks before proceeding to the next phase. Progression through these cell cycle checkpoints is tightly linked to feedback received from damage-seeking signaling pathways in order to protect DNA from *de novo* mutations and erroneous replication [50]. When errors are detected, the default cellular response is to delay or abort zygotic progression in order to assess the issue, and when possible to repair the damage (reviewed by [51]). In our study, depletion of core histones from the mouse PT appeared to have triggered this damage control response, resulting in significantly reduced rates of cleavage and blastocyst formation in histone-deficient zygotes relative to ICSI and sonication controls.

Our findings align with previous reports of issues in male chromatin remodeling and early zygotic development resulting from nuclear protein deficiency. In an experiment that induced double-stranded breaks in the male genome via ionizing radiation of sperm cells, zygotes produced by in vitro fertilization experienced a delay in the first round of DNA replication [52]. Spermatozoa from mice with a transition protein knockout [53] and spermatozoa from chimeric mice with a protamine deficiency [54] share common morphological characteristics, such as reduced nuclear condensation states and elevated levels of DNA strand breaks. ICSI with protamine-deficient spermatozoa resulted in normal levels of oocyte activation, but a significant failure to develop to the blastocyst stage. Inadequate amount and localization of protamine 1 was also found to be associated with defects in bull sperm chromatin structure, coinciding with reduced fertility in bulls [55]. Taken together, these studies provide evidence of altered spermiogenesis and early zygotic development in nuclear-protein-deficient germ cells. Although the mechanism responsible for developmental delay in histone-deficient zygotes remains to be determined, we believe that the bioavailability of PT-derived histones in the ooplasm is inadequate to protect the paternal chromatin as it decondenses. As a result, the net charge of the chromatin, as well as the stoichiometric ratio of its major components, are shifted from being physiologically normal and the chromatin becomes biochemically unstable [54]. The sperm DNA may, thus, become more susceptible to subsequent degradation in the enzymatically active maternal ooplasm.

In order to determine whether DNA instability contributes to a delay in zygotic progression in PT histone deficient zygotes, future work should consider employing TUNEL (terminal deoxynucleotidyl transferase dUTP nick end labeling) to detect DNA fragmentation in the paternal pronuclei. By fluorescently labeling the 3′-hydroxyl termini in DNA strand breaks, this assay could be a useful tool in assessing the degree of damage sustained by the paternal chromatin following ICSI with histone-deficient spermatozoa. If paternal pronuclei were to display higher levels of TUNEL labeling than maternal pronuclei, this would directly support the hypothesis that PT-derived histones protect the paternal chromatin from damage upon entry into the ooplasm. In addition, 5-ethynyl-29-deoxyuridine (EdU) assays could be employed to determine whether DNA replication is delayed in these histone-deficient zygotes. In a study by Gawecka et al. [56], ICSI-generated zygotes injected with spermatozoa that had chromatin fragmentation were assessed 8 h post injection using EdU assays and showed impaired DNA synthesizing activity, but were able to recover in the following hours. Confirmation of the function of PT core histones would represent an important development in our current understanding of the mechanisms at play during early embryogenesis. Given the growing issue of male infertility [57], PT core histones could potentially become valuable biomarkers of sperm quality.

This study has confirmed the presence of histones as prominent constituents of the murid PT, expanding the list of eutherian species (i.e., bovine, porcine, and human) that possess these traditionally nuclear proteins in this non-conventional extra-nuclear sperm head compartment. The localization of primarily core histones to the PT sub-regions that are also assembled from presumed *de novo* histones translated towards the end of spermiogenesis suggests that the PT histones play a significant role in sexual reproduction. This proposition was supported by our ICSI trials in which mouse oocytes fertilized with PT-histone-deficient sperm frequently failed to reach specific embryonic developmental milestones within the standard accepted timeframe for the species. These results lead us to propose that PT core histones exert nuclear stability during paternal pronucleus formation. In other words, the failure of these histone-deficient zygotes to progress at the same rate as controls may be attributable to destabilization and subsequent degradation of the paternal chromatin following its incorporation into the ooplasm. 

## 4. Materials and Methods

### 4.1. Animals

Protocols for animal handling and treatment procedures (protocol #1742) were reviewed and approved by the University Animal Care Committee (UACC) of Queen’s University, in accordance with the guidelines of the Canadian Council on Animal Care (CCAC). All mouse procedures were performed using CD-1 (female and male) or C57BL/6 (male) mice, aged 6–12 weeks, purchased from Charles River Laboratories (St. Constant, QC, Canada). Rat spermatozoa were collected from Sprague–Dawley rats, aged 6–12 weeks, purchased from Charles River Laboratories (St. Constant, QC, Canada) or donated by the Department of Biomedical and Molecular Sciences at Queen’s University. Bovine epididymides used for sperm collection were collected from mature bulls immediately after slaughter from local abattoirs.

### 4.2. Sperm Collection

Spermatozoa were obtained from the fresh cauda epididymides of sacrificed mature rats and mice. The epididymides were placed into phosphate-buffered saline (PBS) or Tris-buffered saline (TBS) solutions (pH 7.4–7.8), depending upon the subsequent methodology. Spermatozoa were collected by cutting and gently squeezing the cauda epididymides so that the spermatozoa diffused into the solution. The sperm-containing suspension was aliquoted into Eppendorf tubes and washed by centrifugation in the buffer of choice at 1000× *g* for 5 min to be used for Western blotting or immediately fixed in 2% formaldehyde before being stored at 4 °C in PBS for use in indirect immunofluorescence. Sperm concentration was determined by counting cells using in a hemocytometer. Intact bovine sperm were extracted according to methods previously described by Oko and Maravei [16] from fresh epididymides or frozen whole epididymides that were thawed. 

For testicular smears, spermatogenic cells were obtained from the testes of sacrificed mature rats and mice. The testicular smears were processed for indirect immunofluorescence according to previously published protocols [58]. Briefly, the tunica albuginea was removed from the testes and the testes were placed in 5ml 200mM PBS. The testes were then cut into smaller pieces using a razor blade and spermatids were allowed to diffuse out of the seminiferous tubules into the solution.

### 4.3. SDS and High-Salt Extraction of Sperm Histones

For SDS extraction, 2 × 10^7^ murid sperm were incubated in 200 µL 2% sodium dodecyl sulfate (SDS) for 5 min at 100 °C. For high-salt extraction, 2 × 10^7^ murid sperm cells were suspended in 200 µL 1 M potassium chloride (KCl) solution and sonicated on ice for 5 s at 0.1 A. Each solution resulting from one of the above extraction methods was promptly separated into supernatant and pellet fractions by centrifugation at 16,000× *g* for 4 min. Following removal of the supernatant, the pellet was washed with PBS to remove any residual solubilized proteins or salts. For storage, the proteins in the KCl supernatant were isolated by chloroform–methanol precipitation and dried. The core histone identity of extracted proteins was verified using SDS-PAGE and Western blot analyses, as described below.

### 4.4. SDS-PAGE and Western Blot Analysis

Immediately following extractions (see above), supernatants and pellets were incubated in reducing sample buffer (200 nM Tris pH 6.8, 4% SDS, 0.1% bromophenol blue, 5% β-mercaptoethanol, 40% glycerol) to preserve their total protein profiles. Samples were separated along a molecular weight (MW) gradient by sodium dodecyl sulfate–polyacrylamide gel electrophoresis (SDS-PAGE), as previously described by Laemmli [59]. A BLUeye pre-stained protein ladder (GeneDirex, Taiwan) and calf thymus histones (Sigma-Aldrich, St. Louis, MO, USA, H9250) were run in adjacent lanes and served as general and histone-specific MW references, respectively. Gels were stained with Coomassie Brilliant Blue 250 (Sigma-Aldrich, St. Louis, MO, USA) or used for immunoblot analysis. For immunoblots, one sample (i.e., sperm supernatants, pellets, and whole sperm) equivalent to 5–10 million sperm was loaded per lane in each experiment and resolved on 4% stacking and 18% separating polyacrylamide. Electrophoresis-separated proteins were transferred onto methanol-activated polyvinylidene fluoride (PVDF) membranes (Millipore, Mississauga, ON, Canada) according to methods adapted from [60]. To prevent non-specific labeling, membranes were blocked with 10% skim milk in PBS containing 0.05% Tween-20 (PBS-T). Membranes were then cut at the 25 kDa level for probing with two separate primary antibodies at 4 °C, overnight; the upper PVDF strip (>25 kDa) was incubated in anti-PAWP serum (1:1000 dilution), while the lower strip (<25 kDa) was incubated in anti-pan-histone serum (1:4000 dilution). Membranes were washed generously with PBS-T before and after incubation with an HRP-conjugated secondary antibody (diluted to 1:40,000). The resulting blots were exposed to Clarity™ Western ECL Substrate (Bio-Rad, Mississauga, ON, Canada) and developed on X-ray film (Eastman Kodak Company, Rochester, NY, USA).

### 4.5. Antibodies

The primary antibody used for Western blotting and immunodetection experiments was a polyclonal rabbit anti-histone made in house against purified bovine calf thymus histones (anti-pan histone antibody, also rereferred to as H5). Antibodies specific to each of the histone subtypes were subsequently made through affinity purification of the original serum using recombinant forms of each histone [29]. An affinity purified polycolonal rabbit H3 specific antibody (Proteintech, Rosemont, IL, USA, 17168-1-AP) and a testis-specific H2B antibody (TH2B) (EMD Millipore, Burlington, MA, USA, 07-680) were also used at the manufacturer’s suggested concentrations in all applications. The anti-pan histone (H5) antibody was applied at a 1:4000 concentration for Western blot analysis and at a 1:50 concentration for immunofluorescence and immunoperoxidase histochemistry. For Western blot analysis, the secondary antibody used was goat anti-rabbit IgG-HRP (horseradish peroxidase) (VectorLab, Burlingame, CA, USA, 1:50,000), whereas for immunofluorescence, donkey anti-rabbit IgG-CFL488 (Santa Cruz, Dallas, TX, USA sc-362261, 1:200) was used. The secondary antibody used in enzymatic immunohistochemistry was a biotin-labeled anti-rabbit IgG (VectorLab, Burlingame, CA, USA 1:200). A mouse monoclonal anti-alpha tubulin antibody (Sigma-Aldrich, St. Louis, MO, USA, T6074) was used in immunofluorescence co-localization experiments at a concentration of 1:2500 and a donkey-anti mouse IgG-CFL647 (Santa Cruz, Dallas, TX, USA sc-362288 1:200) was used as the secondary antibody.

### 4.6. Immunofluorescence

Histones of whole or protein-extracted rat and mouse spermatozoa were analyzed by indirect immunofluorescence according to methods described by Sutovsky et al. [61]. Spermatids or spermatozoa were mounted on poly-L-lysine-coated coverslips in KMT buffer (100 mM KCl, 2 mM MgCl_2_, 10 mM Tris-HCl, 5 mM EGTA, pH 7.0). The cells were fixed with 2% formaldehyde in phosphate saline buffer (PBS) for 30 min before being permeabilized in PBS with 0.1% Triton-x-100 (PBS-Tx) for 40 min at room temperature. The cells were blocked with 5% normal goat serum (NGS) in PBS-Tx for 25 min to avoid non-specific binding, then incubated in a humidity chamber with primary antibody diluted in 1% NGS overnight at 4 °C. On the following day, the cells were washed with 1% NGS three times followed by a 40 min incubation with fluorescently tagged secondary antibodies diluted in 1% NGS at room temperature and shielded from light. The secondary antibody mixture included blue fluorescence DNA stain DAPI (4′,6-diamidino-2-phenylindole, dihydrochloride). The coverslips were then mounted onto glass slides using Vectashield mounting media (Vector Laboratories, Burlingame, CA, USA) and sealed with nail polish. Images were captured at the Queen’s University Cancer Biology Institute Imaging Centre using a Quorum Wave Effects spinning disc confocal microscope.

### 4.7. Immunohistochemistry

Murid testicular sections from testes that had been perfusion-fixed in Bouin’s fixative or paraformaldehyde and embedded in paraffin were deparaffinized in xylene and hydrated through a graded series of ethanol solutions. During hydration, the sections were treated to abolish endogenous peroxidase activity, to neutralize residual picric acid, and to block free aldehyde groups [22]. Once hydrated, the sections were subjected to antigen retrieval by microwave irradiation in a 5% urea Tris-HCl, pH 9.5 solution. Immunolabeling was conducted using an avidin-biotin complex (ABC) kit (Vector Laboratories, Burlingame, CA, USA) and followed the procedure outlined by Ferrer et al. [62].

### 4.8. Electron Microscopic Immunocytochemistry

Adult male Sprague–Dawley rats (350–450 g) were anesthetized and their testes were fixed by perfusion for 10 min through the abdominal aorta with 0.5% glutaraldehyde and 4% paraformaldehyde in 0.1 M phosphate buffer containing 50 mM lysine at pH 7.4. After their removal, the testes were cut into small pieces (0.5 mm^3^), immersed for 2 h in the above fixative at 4 °C, washed 2–3 times in PBS containing 4% sucrose (pH 7.4), and treated with PBS containing 4% sucrose and 50 mM NH_4_Cl for 1 h at 4 °C. The tissue pieces were washed, dehydrated in a graded series of methanol up to 90%, and embedded in LR white (Canemco, St Laurens, QC, Canada). They were then polymerized at −20 °C, cut into ultrathin sections, and mounted on Formavar coated nickel grids. During immunolabeling, grids were floated tissue-side down on drops of various solutions. Sections were blocked with 5% NGS in TBS, then incubated with affinity purified anti-histone antibodies overnight at 4 °C. They were subsequently washed in TWBS (TBS with 0.1% Tween-20, pH 8) extensively, blocked with 5% NGS for 15 min, incubated in 10 nm gold-particle-conjugated goat anti-rabbit IgG (Sigma, Mississauga, ON, Canada) for 2 h at 21 °C, washed with TWBS and deionized water, and allowed to dry. The sections were then counterstained with uranyl acetate and lead citrate, washed with deionized water, and dried. Photographs were taken using a Hitachi 7000 transmission electron microscope. The results shown are typical of three different animals and experiments.

### 4.9. LC-MS/MS and Data-Dependent Aquisition (DDA)

Precipitated proteins of the sperm KCl extracts (see Methods above) were resuspended in rehydration buffer (8 M Urea, 2% sodium deoxycholate, 10 mM Tris, pH 7.5). To the suspension, 10 mM DTT was added for 1h, followed by 20 mM iodoacetamide for another hour in the dark. In order to precipitate the sample, one volume of chloroform and two volumes of methanol were added to two volumes of sample and the mixture was vortexed vigorously. This sample was centrifuged (10,000× *g*, for 2 min), then after phase separation, the upper phase was removed. Three volumes of methanol were added to the remaining lower phase and the mixture was gently inverted twice. The solution was then centrifuged (10,000× *g*, 15 min) to pellet the precipitated protein. The supernatant was discarded and the pellet was allowed to air dry. Trypsin (1:50, *w*/*w*) was added to the pellet and digested overnight at 37 °C. Samples were centrifuged (17,000× *g* for 20 min), transferred to sample vials, and put under vacuum concentration until dry.

NanoLC-MS/MS was performed using a Dionex UltiMate 3000 nanoLC system (Dionex). Peptides from the trypsin-digested samples of the histone-enriched PT fraction were suspended in buffer A (2% ACN/0.1% TFA) and directly loaded onto a 50 cm analytical column packed with Acclaim PepMap C_18_ 2 μm sorbent. Peptides were eluted using a 110 min gradient from 7 to 40% buffer B (95% ACN, 0.1% TFA) at 250 nL min^−1^ and nanoelectrosprayed into a Q-Exactive Plus (Thermo Fisher Scientific, Waltham, MA, USA). Precursor scans of intact peptides were measured in the Orbitrap by scanning from *m*/*z* 350 to 1500 (with a resolution of 70,000), then the fifteen most intense multiple charged precursors were selected for HCD fragmentation with a normalized collision energy of 32.0 and measured in the Orbitrap at a resolution of 35,000. Automatic gain control targets were 3^E6^ ions for Orbitrap scans and 5^E5^ for MS/MS scans. Dynamic exclusion was employed for 15 s. Fragmentation data were converted to peak lists using Xcalibur version 4.027.19 (Thermo Fisher Scientific) and the HCD data were processed using Proteome Discoverer 2.1 (Thermo Fisher Scientific, Waltham, MA, USA). MS spectra were then searched with Mascot V2.6 (accessed on 5 December 2019) against all mouse and rat entries in SwissProt (Release December 2019, 17,033 entries). Mass tolerances in MS and MS/MS modes were 10 ppm and 0.02 Da, respectively; trypsin was designated as the digestion enzyme and up to two missed cleavages were allowed. Carbamidomethylation of cysteine residues was designated as a fixed modification. The variable modifications included were oxidation of methionine and deamidation of asparagine or glutamine. Interrogation of the corresponding reversed database was also performed to evaluate the false discovery rate (FDR) of peptide identification using Percolator based on q-values, which were estimated from the target–decoy search approach. To filter out target peptide spectrum matches (target-PSMs) over the decoy PSMs, a FDR of <1% was set at the peptide level. An additional identification criterion sought a minimum of two uniquely matched peptides per protein.

### 4.10. Separation of Testicular Germ Cells from Rat Testes Using Unit Gravity Sedimentation

The enzymatic digestion of testicular cells and their sedimentation on a BSA gradient using a STAPUT apparatus were assessed using an approach adapted from previous publications on this method [63,64,65].

Two Sprague–Dawley rats were asphyxiated with CO_2_ following the standard operating procedures of the Queen’s Animal Care Committee. Testes were retrieved, decapsulated, and separated into two 50mL tubes containing 30mL of RPMI-1640 medium (Hyclone, Logan, UT, USA, SH30011.03) with 1.5 mg/mL collagenase (Bioshop, Burlington, ON, Canada COL007.500). The tubes were incubated for 30 min at 37 °C with agitation. The separated and digested testicular tubules were collected by centrifugation at 200× *g* for 5 min at 4 °C, then washed once with RPMI-1640 medium. Once the supernatant was removed, the tubules were cut into pieces, mixed with 30 mL of preheated RPMI-1640 medium containing 1% BSA pH 7.4 (Fisher Bioreagent, Waltham, MA, USA, S-15898) and 1.5 mg/mL trypsin (Gibco, Waltham, MA, USA, (2.5%) #12090), and incubated for 20 min at 37 °C with agitation. Then, 10% FBS (ThermoFisher Scientific, Waltham, MA, USA) was added to the above mixture, supplemented by 15 uL/tube (150U) of DnaseI (Bioshop, Burlington, ON, Canada, DRB001.10) to prevent high viscosity due to genomic DNA. Tubular cells were released by performing gentle pulsing movements with a transfer pipette for 3–5 min. Subsequently, the cells were filtered through a 100 μm cell strainer. 

Using a STAPUT sedimentation apparatus set up in a refrigerated room with minimal vibration, the testicular cell suspension was loaded on a 2–4% BSA gradient at pH 7.4. The flow rate was adjusted to form the gradient in 70 min. The cells were then placed on the gradient and allowed to flow through for 90 min. The first five 10 mL fractions that were collected were made up of unwanted debris. After this initial collection, the circuit was closed to allow for another 90 min of cell separation. Fractions of 10 mL were then collected at a rate of 10 mL/30 min and their purity levels for tetraploid cells and round spermatids were assessed and pooled accordingly. For purity analysis, cells were fixed in Carnoy’s fixative(methanol/acetic acid 3:1) and stained with DAPI or periodic acid–Schiff stain. The cellular perimeter and cellular surface of the tetraploid cells and haploid cells were assessed using phase microscopy. Cells in each pool were counted using a hemocytometer and aliquots of 1 × 10^6^ cells/tube were compiled for RNA analysis.

### 4.11. RNA Isolation and Quantitative PCR (RT-qPCR) Analysis of Somatic H2B mRNA

The RNA was extracted using a combination of TRIZOL (Invitrogen, Waltham, MA, USA, 15596-018) and the Tissue Total RNA Mini Kit (GeneAid, Taiwan, RT050). Once the TRIZOL extraction was completed, following the manufacturer’s recommendations, one volume of 70% ethanol was added to the aqueous phase and loaded on an RB column. Subsequent steps were performed per the manufacturer’s recommendation. Triplicates of RNA extractions were processed for each group, since there were no reliable reporter genes that could have been used to measure the relative expression of H2B in a population of tetraploid cells compared to round spermatids. 

RNA samples were digested with DNAseI, then rat testicular and somatic H2B mRNA samples were reversed-transcribed using a High-Capacity cDNA Reverse Transcription Kit (Applied Biosystems, Waltham, MA, USA, #436884) according to the manufacturer’s instruction. The RT primer used was 5′-GCTGGTGTACTTGGTG-3′, which is specific to both somatic and testicular H2B. Proper controls were prepared to ensure that each sample was genomic-DNA-free. Somatic H2B qPCR primers were designed from Rattus H2B (NCBI; NM_022647.2), making sure that they were not going to amplify the testis-specific variant H2B (GenBank: M18046.1). The forward primer was 5′-ATGCCTGAGCCTGCGAAGT-3′, while the reverse primer was 5′-CATGGCCTTGGAAGAGATGC-3′.

The relative mRNA expression of rat somatic H2B was measured using Roche LightCycler^®^ 480 System II (Roche Scientific, Laval, QC, Canada). The cycling parameters consisted of an initial denaturation step at 95 °C for 10 min, then another 20 s in the same conditions, followed by touchdown annealing temperatures from 70 to 67 °C (decreasing step size of 0.5 °C per cycle) for 15 sec each and 72 °C for 15 s to ensure precise hybridization. The total number of cycles was 43. Finally, melt curve analyses were performed to ensure the quality and specificity of qPCR products.

### 4.12. Gamete Isolation for Intracytoplasmic Sperm Injection

Oocyte collection: Mature CD-1 female mice aged 8–12 weeks were superovulated with intraperitoneal injections of 10 IU pregnant mare serum gonadotropin (PMSG; Sigma-Aldrich, St. Louis, MO, USA) and 10 IU human chorionic gonadotropin (hCG; Sigma-Aldrich, St. Louis, MO, USA), administered 48 h apart. Mice were euthanized at 12–14 h post-hCG injection by isoflurane inhalation and cervical dislocation and oviducts were removed and placed in Advanced KSOM (Sigma-Aldrich, St. Louis, MO, USA, MR-101-D). Cumulus-oocyte complexes were released and transferred to Advanced KSOM droplets containing 0.1% hyaluronidase (Sigma-Aldrich, St. Louis, MO, USA, H3757) for cumulus cell removal. Cumulus-free oocytes were washed and incubated in Advanced KSOM with mineral oil (Sigma-Aldrich, St. Louis, MO, USA, M8410) at 37 °C and 5% CO_2_ until use.

*Sperm collection*: Study animals were euthanized by isoflurane inhalation and cervical dislocation. Mouse cauda epididymides were isolated from adult males, pierced with a needle, and gently squeezed to allow the sperm to diffuse into a droplet of human tubal fluid (HTF) (Millipore, Burlington, MA, USA MR-070-D) insulated by mineral oil (ICSI CTRL) or into PBS for subsequent treatment. For high-salt extraction, sperm was spun down and resuspended in 200 µL 1 M potassium chloride (KCl) solution and sonicated on ice for 5 s at 0.1 A (histone-depleted, HD), whereas the sonicated control (SON CTRL) was sonicated on ice for 5 s at 0.1 A in PBS. The sperm were subsequently incubated at 37 °C and 5% CO_2_ until required, no more than three hours post-collection. In each ICSI replicate, spermatozoa from one male were used for all treatments to control for variations between animals.

### 4.13. Intracytoplasmic Sperm Injection (ICSI)

ICSI was carried out with CD-1 mice according to the methods described by Yanagimachi [66] across 3–4 experimental trials. The procedure was performed using a Nikon Ti-S inverted microscope (Nikon Canada Inc., Mississauga, ON, Canada) fitted with Narishige micromanipulators (Narishige International US Inc., Amityville, NY, USA) and a Piezo PMM150HJ/FU drill (Prime Tech Ltd., Ibaraki, Japan). Briefly, a single spermatozoon was drawn up into the injection pipette from a sperm suspension containing 6–10% polyvinylpyrrolidone (PVP; Sigma-Aldrich, St. Louis, MO, USA, PVP360). Motile sperm were immobilized by applying several piezo pulses to the midpiece, separating heads and tails. Injections were performed in Advanced KSOM droplets; the oocyte was held in place by the holding pipette and piezo pulses were applied to “drill” through the zona pellucida with the injection pipette. Once the zona was punctured, the sperm head was expelled into the ooplasm near the opposite side of the oocyte and the pipette was gently withdrawn. Sperm-injected oocytes were left to rest at RT for 10 min before being transferred to new Advanced KSOM droplets under mineral oil at 37 °C, 5% CO_2_. Injected embryos were left to incubate for 6 h, 22–24 h, 4 d, or 5 d, depending on experimental group. Oocytes were analyzed by differential interference contrast microscopy on a Nikon Ti-S inverted microscope (Nikon Canada Inc., Mississauga, ON, Canada) and photographed using a QImaging QICAM Fast 1394 digital camera.

### 4.14. Statistics

Statistical significance was determined based on two-tailed T-tests performed between ICSI CTRL, SON CTRL, and histone-depleted (HD) experimental groups. The standard error of the mean was calculated to reflect variation within each group. Here, *p* values < 0.05 were considered significant.

## Figures and Tables

**Table 1:** Comparison of mass spectrometry analyses of PT histones between species.

Histone	Species						
H1	Mouse	H1.1^canonical^	H1.4^canonical^				H1LS1^testis^
	Rat	H1.1^canonical^	H1.4^anonical^	H1.5^canonical^		H1.0^canon/variant^	H1T^testis^
	Bull						
H2A	Mouse	H2A^core^	H2A 2-A^core^	H2A 2-B^core^	H2A 2-C^core^	H2AX^core/variant^	H2AV^variant^
	Rat	H2A^core^	H2A 2^core^				H2AV^variant^
	Bull				H2A 2-C^core^		H2AV^variant^
H2B	Mouse	H2B 2-B^core^					H2B 1-A^testis (TH2B)^
	Rat	H2B^core^	H2B 1N^core^				H2B 1-A^testis (TH2B)^
	Bull		H2B 1N^core^				
H3	Mouse	H3^core^				H3.3^core/variant^	
	Rat	H3^core^				H3.3^core/variant^	
	Bull	H3^core^				H3.3^core/variant^	
H4	Mouse	H4^core^					
	Rat	H4^core^					
	Bull	H4^core^					

Note: ^core^ canonical histones incorporated into nucleosome whose synthesis is usually DNA replication dependent; ^variant^ substitute for core or linker histones in nucleosomes and can confer specific structural/functional features; ^core/variant^ can play the role of both and be incorporated in either a replication dependent or independent manner; ^testis^ histone specific to the testes; ^canonical^ conventional linker protein found in somatic tissue whose synthesis is usually DNA-replication-dependent; ^H2A−2C^ H2ac20.

**Table 2:** Comparison of mass spectrometry analyses of PT histones between species.

Species	Histone	Post-Translational Modifications
Mouse	H1.1	K18me2, K21me, K22me
	H2B 2-B	K17ac, K21ac
	H3.1	K18ac, K23ac, R27me2, K27me2, K79me2
	H3.3	K18ac, K23ac, K27ac, K79ac, K36me, K37me, K79me, R26me2, K27me2, K36me2,K37me2, K79me2
	H4	K20me, R23me2
Rat	H3.1	K23ac, K79ac, K36me, K37me, K79me, K27me2, K36me2,K37me2, K79me2, K27me3
	H3.3	K23ac, K79ac, K36me, K37me, K79me, K27me2, K36me2,K37me2, K79me2, K27me3

## Supplementary Materials

The following are available online at https://www.mdpi.com/article/10.3390/ijms22158119/s1.
