# Supplementary material for: Core Histones Are Constituents of the Perinuclear Theca of Murid Spermatozoa: An Assessment of Their Synthesis and Assembly during Spermiogenesis and Function after Gametic Fusion
Source: Int J Mol Sci. 2021 Jul 29;22(15):8119. doi: 10.3390/ijms22158119 (PMC8347300; doi:10.3390/ijms22158119)
Supplement: Supplementary file 1 [file ijms-22-08119-s001.zip › ijms-1308579-supplementary.pdf]

Supplementary Figures

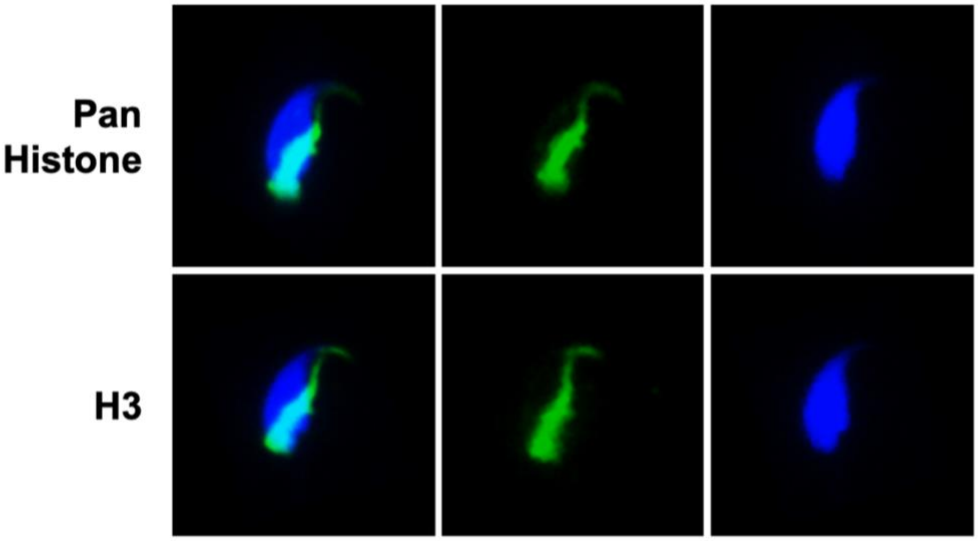

Figure S1. Histone localization in mouse spermatozoa. Mouse spermatozoa probed with a pan-histone antibody (PAN) that reacts to all core histones and with a H3 affinity purified antibody (H3). Reactivity pattern (green) is merged with nuclear DAPI labeling (blue). Note the continuity in immunolabeling between the perforatorium and postacrosomal sheath of the PT. Bars = 10 μm.

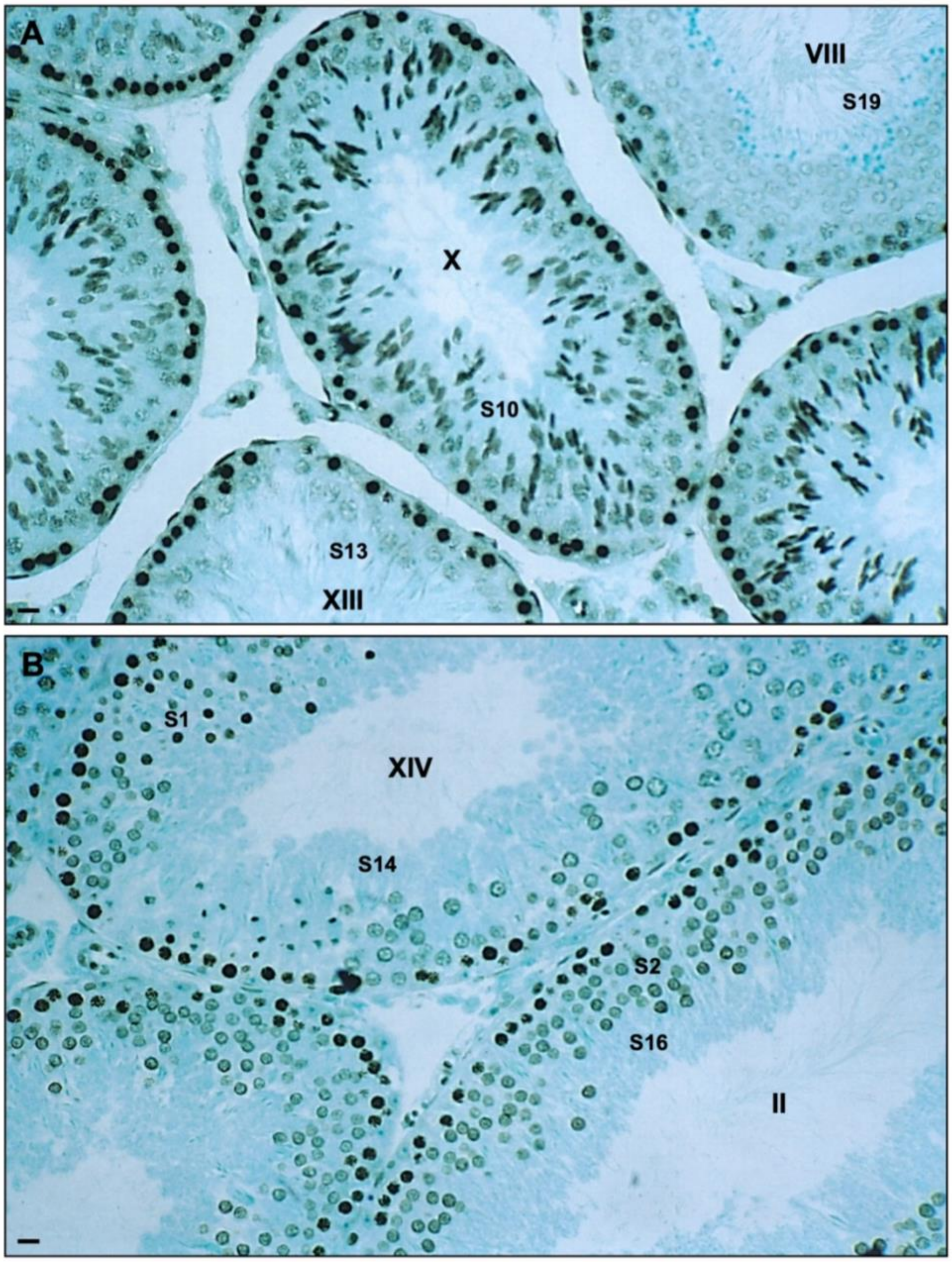

Figure S2. Light micrographs of immunoperoxidase-labeled sections through rat seminiferous tubules of stages II, VIII, X, XIII, and XIV of the cycle probed with affinity-purified α-H3 antibody. (**A**) Nuclear histone immunoreactivity of spermatids disappears in step 13 spermatids (S13) in stage XIII. (**B**) In stages XIV and II histone labelling in step14 (S14) and 16 (S16) spermatids is undetectable at this magnification. Bar = 5 μm; S2 – S19, spermatid steps 1 to 19 of spermiogenesis.

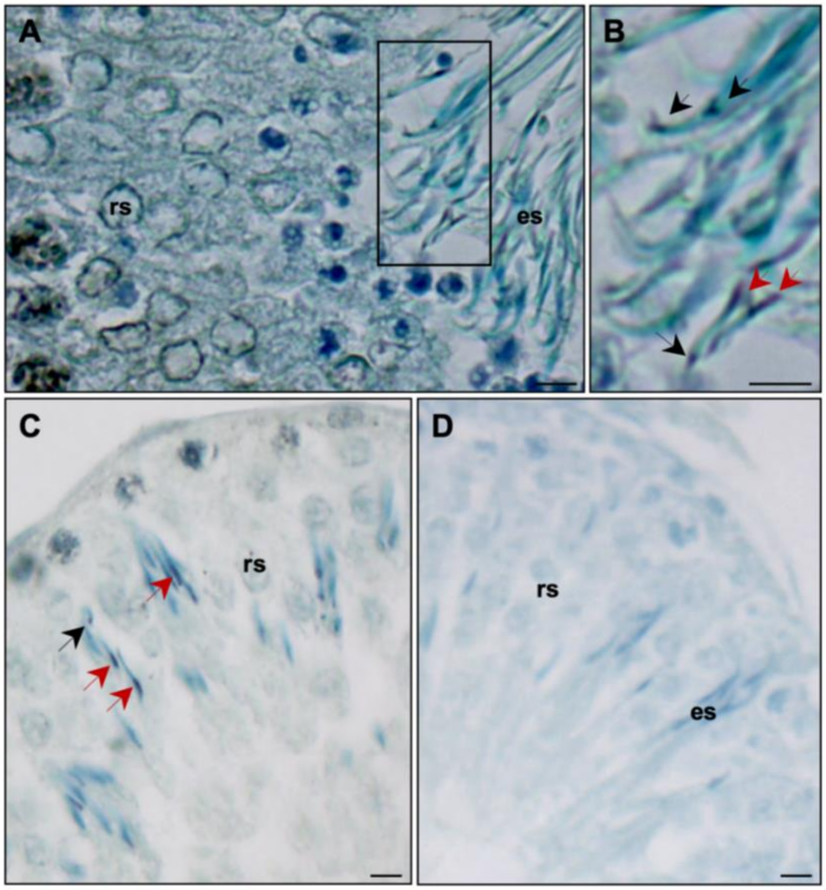

Figure S3. High magnification light micrographs of immunoperoxidase-labeled sections through seminiferous tubules of stages VIII and III probed with an affinity-purified α-H3 antibody. (A) Step 19 spermatids undergoing spermiation. Note the mature step 19 spermatids (in designated boxed area) magnified to advantage in inset (B) Immunoreactivity to the antibody in the regions of the perforatorium (black arrows) and postacrosomal sheath (red arrows) of the PT. The perforatoria display the characteristic triangular rod like structure. C. Stage III displaying immunoreactivity in the postacrosomal sheath (red arrows) of step 16 spermatids. The immune-reactive perforatorium can occasionally be detected (black arrow). D. Stage III seminiferous epithelium control section probed with pre-immune serum from rabbit that produced anti-sera to the four histones. Note that no reactivity is evident. Bars = 5 μm.

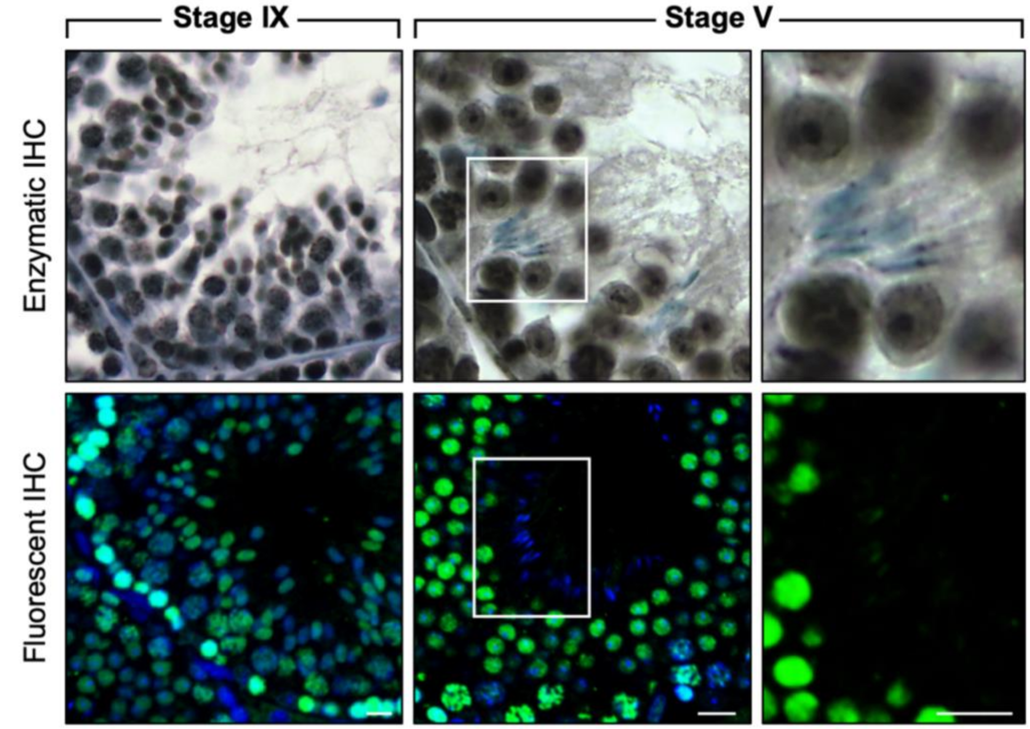

Figure S4. Light micrographs of immunoperoxidase- and fluorescent-labelled sections through mouse seminiferous tubules of stages IX and V probed with affinity-purified α -H3 antibody. In stage IX, the nuclei of all germ cells are immuno-reactive including the nuclei of elongating spermatids in step 9 of spermiogenesis. However, in stage V only the caudal and apical portions of the step 17 elongated spermatid heads (see magnified insets) are reactive, the antigenicity most likely arising from the postacrosomal and perforatorial regions of the PT. Bars = 10 μm.

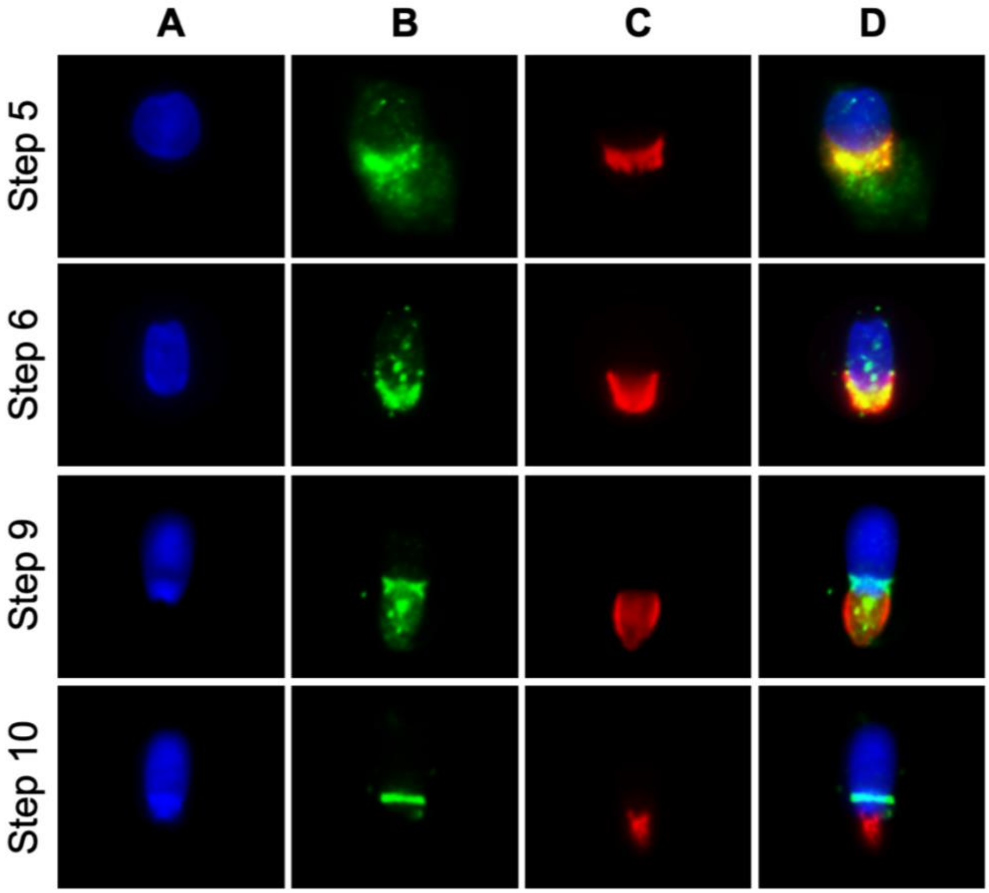

Figure S5. Immunofluorescent micrographs of the co-localization of the Pan α-Histone (green) and α-tubulin (red) antibodies on the manchette of elongating porcine spermatids. Boar spermatids from testicular extracts were fixed in 2% formaldehyde and permeabilized after fixation with Triton-X-100. (**A**) DAPI alone. (**B**)Anti-histone antibody alone. (**C**) Anti-tubulin antibody alone. (**D**)Panels A, B, and C merged. Note that in the eleven spermatid steps of swine spermiogenesis the histones assemble as part of the PAS (arrow) in step 9, in the wake of the descending manchette. By step 10 the PAS is completely formed and the descended manchette begins to dissemble. Blue = DAPI, Green = anti-pan histone, Red = anti-tubulin, Yellow = co-localization of the histones and tubulin. Bars = 5 μm.

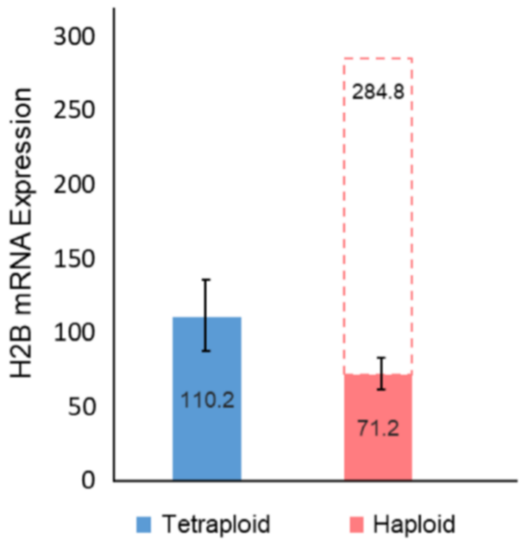

Figure S6. Quantitative PCR analysis measuring the expression of somatic H2B mRNA in round (haploid) spermatids (red) *vs.* tetraploid cells (blue). Based on same number of haploid and tetraploid cells, results are expressed as a function of cell number. However, if the results are adjusted based on DNA quantity (tetraploid cells have 4x more DNA than haploid cells) it is estimated that the haploid cell has 2.6 times more somatic H2B per DNA (indicated by dashed region in graph) than the tetraploid cell.
